# Predicted Value of MicroRNAs, Vascular Endothelial Growth Factor, and Intermediate Monocytes in the Left Adverse Ventricular Remodeling in Revascularized ST-Segment Elevation Myocardial Infarction Patients

**DOI:** 10.3389/fcvm.2022.777717

**Published:** 2022-03-24

**Authors:** Raquel Del Toro, Isabel Galeano-Otero, Elisa Bevilacqua, Francisco Guerrero-Márquez, Debora Falcon, Agustín Guisado-Rasco, Luis Díaz-de la Llera, Gonzalo Barón-Esquivias, Tarik Smani, Antonio Ordóñez-Fernández

**Affiliations:** ^1^Departamento de Fisiología Médica y Biofísica, Universidad de Sevilla, Seville, Spain; ^2^Grupo de Fisiopatología Cardiovascular, Instituto de Biomedicina de Sevilla-IBiS, Universidad de Sevilla/HUVR/Junta de Andalucía/CSIC, Seville, Spain; ^3^Servicio de Cardiología, Hospital Universitario Virgen del Rocío, Seville, Spain

**Keywords:** STEMI, PPCI, inflammatory cells, monocytes, left ventricular adverse remodeling

## Abstract

**Background:**

Primary percutaneous coronary intervention (PPCI) in patients with ST-segment elevation myocardial infarction (STEMI) improves the survival of patients; nevertheless, some patients develop left ventricular adverse remodeling (LVAR) a few months after the intervention. The main objective of this study was to characterize the role of pro-inflammatory cell populations, related cytokines, and microRNAs (miRNAs) released after PPCI as reliable prognostic biomarkers for LVAR in patients with STEMI.

**Methods:**

We evaluated the level of pro-inflammatory subsets, before and after revascularization, 1 and 6 months after PPCI, using flow cytometry. We also performed a miRNA microarray in isolated peripheral blood mononuclear cells (PBMCs) and examined the levels of 27 cytokines in patients’ serum of patients by multiplex ELISA.

**Results:**

We observed that the levels of classical and intermediate monocytes increased 6 h after PPCI in patients who developed LVAR later. Multivariate regression analysis and ROC curves indicated that intermediate monocytes, after PPCI, were the best monocyte subset that correlated with LVAR. Within the 27 evaluated cytokines evaluated, we found that the increase in the level of vascular endothelial growth factor (VEGF) correlated with LVAR. Furthermore, the microarray analysis of PBMCs determined that up to 1,209 miRNAs were differentially expressed 6 h after PPCI in LVAR patients, compared with those who did not develop LVAR. Using RT-qPCR we confirmed a significant increase in miR-16, miR-21-5p, and miR-29a-3p, suggested to modulate the expression of different cytokines, 6 h post-PPCI in LVAR patients. Interestingly, we determined that the combined analysis of the levels of the intermediate monocyte subpopulation, VEGF, and miRNAs gave a better association with LVAR appearance. Similarly, combined ROC analysis provided high accurate specificity and sensibility to identify STEMI patients who will develop LVAR.

**Conclusion:**

Our data suggest that the combined analysis of intermediate monocytes, VEGF, and miRNAs predicts LVAR in STEMI patients.

## Introduction

During a myocardial infarction with ST-segment elevation (STEMI), the time of coronary occlusion is of vital importance until blood flow is reestablished. Primary percutaneous coronary intervention (PPCI) considerably mitigates cardiac cell death and adverse cardiovascular events (1). The occlusion of the coronary artery initiates multiple structural, functional, and metabolic lesions that negatively affect the function of the heart. Several studies have suggested the beneficial effects of early PPCI on left ventricle remodeling, which demonstrated that significant left ventricular dilation occurred in a relevant proportion of patients with STEMI treated with PPCI (2, 3). Pathological remodeling appears because of progressive change in the left ventricle size, shape, and function, causing left ventricular adverse remodeling (LVAR), which can lead to heart failure (HF) (3). There is a general consensus that acute inflammation and especially the innate immune system influences the clinical outcome in HF patients (1). Immune cells play an important role in protecting the myocardium from ischemic damages and promote wound healing in the ischemia-affected tissues (2). Therefore, the control and expansion of pro-inflammatory populations are considered hallmarks of inflammation resolution. Conversely, excessive production and infiltration of innate cells promote LVAR and increase the probability of suffering adverse cardiac events (3, 4). Hence, given its complexity, it is still unclear how the innate immune system influences the development and progression of LVAR toward HF.

Leukocytes proliferate in the bone marrow (BM) and in response to damage-associated molecular patterns (DAMPs) secreted by tissue, they egress from the BM in order to infiltrate the ischemic myocardium (5). These inflammatory cell populations synthesize a broad range of pro-inflammatory mediators as chemokines, cytokines, microRNAs (miRNAs), and resolution factors, which are key regulators of the inflammation in the myocardium after a STEMI (6, 7). Within leukocyte cells, neutrophils have been recognized as the first innate inflammatory cell population to infiltrate the heart following an acute myocardial infarction (AMI) (8), but 3 days after AMI monocytes became the most important inflammatory cells involved in the salvage of the myocardium (4, 9). Monocytes are a heterogeneous cell population divided in subsets with specific functions and phenotypes during the process of inflammation. Three subsets of human monocytes have been immunodefined (10). The differential expression of CD14 (Lipopolysaccharide LPS receptor) and CD16 (the low affinity receptor for IgG, FCγIIIR) distinguishes classical (CD14++ CD16+), intermediate (CD14++ CD16+), and non-classical (CD14+ CD16++) monocytes.

On the other hand, several studies have demonstrated that miRNAs, the most studied small non-coding RNA, are dysregulated in patients with cardiovascular diseases, as compared with healthy patients, some of them predict the LVAR (11). Recently, the expression patterns of circulating miRNAs have been described in patients suffering a myocardial infarction (11) and after a PPCI (12). miRNAs are known to regulate the expression of key proteins involved in the response to AMI (13). However, little is known about their association with changes in the expression of cytokines and the levels of inflammatory cells population, key effectors during AMI responses.

In this study, we analyzed changes in the levels of inflammatory cell subsets during STEMI and after PPCI, and we further correlated their changes with the clinical appearance of LVAR. We also determined miRNAs levels in peripheral blood mononuclear cells (PBMCs) and of pro-inflammatory cytokines in the serum of these patients. Our combined analysis establishes three components that predict the appearance of LVAR in patients with revascularized STEMI.

## Materials and Methods

This study was conducted following the principles published by the declaration of Helsinki and its modification or similar ethical standards. The study was authorized by the local Ethics Committee on Human Research at the University Hospital “Virgen del Rocio” of Seville (Approval no. 2013PI/096). To report our findings, we followed the “strengthening the reporting of observational studies in epidemiology” (STROBE) guidelines (Supplementary Figure 1).

A detailed section of material and methods is provided in Supplementary Information. We recruited a cohort of patients with revascularized STEMI and controls with rigorous inclusion criteria. We evaluated LVAR using echocardiography and extracted patients’ blood samples before revascularization (0 h), and at different time points after PPCI (6 h, 1, and 6 months). We analyzed inflammatory cell populations using flow cytometry; and serum cytokines levels using Multiplex and ELISA assays. miRNAs expression was examined by miRNA array assay and RT-qPCR.

## Results

### Study of the Cohort Clinical Data

Demographical and clinical information were obtained from 28 healthy controls and 44 patients with STEMI who underwent PPCI. Healthy controls (46 ± 11 years; 57.14% male sex) were patients who did not suffer from arterial hypertension, dyslipidemia, or type II diabetes mellitus and did not smoke, suggesting a cohort without apparent cardiovascular risk. Within the STEMI patient group (58 ± 10 years; 92.10% male sex), 36.84% have arterial hypertension, 47.37% dyslipidemia, 18.42% diabetes mellitus type II, and 47.37% were smokers.

### Analysis of the Level of Inflammatory Cells in ST-Segment Elevation Myocardial Infarction Patients Undergoing Primary Percutaneous Coronary Intervention

**FIGURE 1:**
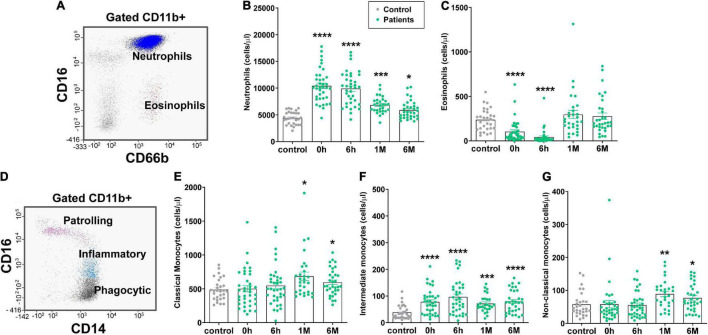
Granulocytes, monocytes and monocyte subsets levels in STEMI patients measured by flow cytometry. **(A)** Flow cytometry strategy with a dot plot gated in CD11b+ cells is used to identify neutrophils (CD11b+, CD16+, CD66b+, blue) and eosinophils (CD11b+, CD16-, CD66b+, red). **(B,C)** Bar graphs show levels of neutrophils **(B,C)** eosinophils in healthy controls (gray dots, *n* = 28) compared with the levels in STEMI patients (green dots, *n* = 38) measured in peripheral blood (PB) at 0 h (before revascularization), 6 h (after revascularization), 1 month and 6 months. **(D)** Representative flow cytometry diagrams of a patient’s PB sample depicting monocyte subsets: non-classical (patrolling, CD11b+ CD14+ CD16++, pink), intermediate (inflammatory, CD11b+ CD14+ CD16+, blue), and classical (phagocytic, CD11b+ CD14+ CD16-, black) monocytes. **(E–G)** Quantification of the level of classical **(E)**, intermediate **(F)** and non-classical **(G)** monocytes in controls and STEMI patients at 0 h, 6 h, 1 month and 6 months after the ischemic event. Values are presented as the means ± SEM. Kruskal–Wallis test with multiple comparisons corrected by Dunn’s test was performed. (*), (**), (***) and (****) indicate significance at *p* < 0.05, *p* < 0.01, *p* < 0.001 and *p* < 0.0001, respectively. PB, peripheral blood; STEMI, ST-elevation myocardial infarction.

Since inflammatory innate cells are key players in the inflammatory response to AMI, we examined by flow cytometry (Figure 1A) the level of CD11b + granulocyte populations (neutrophils and eosinophils) at different time points, before PPCI (0 h), and 6 h, 1, and 6 months after PPCI. Figure 1B shows a high increase in neutrophil levels (neutrophilia) in STEMI patients, as compared with healthy controls. Neutrophilia was observed at the onset and 6 h after PPCI. This increase was mild even if it was still statistically different 1 and 6 months after the intervention. By contrast, the level of eosinophils decreased significantly before and 6 h after PPCI in patients with STEMI, as compared with the control (Figure 1C); while they recovered their basal levels 1 and 6 months after PPCI.

Since monocytes and neutrophils are the main populations egressing from the BM after acute infections or in response to internal injuries as a cardiovascular ischemic event (8), we analyzed the level of monocytes by flow cytometry (Figure 1D) and quantified monocytes subsets populations (Figures 1E–G). As illustrated in Figure 1E, the level of classical monocytes (CD16- CD14++) increased significantly 1 and 6 months after PPCI, but not at the onset neither 6 h after PPCI. Meanwhile, the level of intermediate monocytes (CD16+ CD14++) was significantly increased in STEMI patients before and 6 h after PPCI. This increase lasted up to 6 months, as compared with the healthy controls (Figure 1F). In the case of non-classical monocytes (CD16++ CD14-), we observed only a significant increase 1 and 6 months after PPCI (Figure 1G). Altogether, these data indicate that neutrophils and intermediate monocytes could be involved in the acute inflammatory responses after PPCI in STEMI patients.

### Levels of Inflammatory Cells in ST-Segment Elevation Myocardial Infarction Patients With Left Ventricular Adverse Remodeling

In order to analyze the number of STEMI patients who developed LVAR after PPCI, we performed an echocardiography of all the patients at the hospital discharge and 6 months after the ischemic event. Table 1 shows that 12 of 38 patients (31.58%) developed LVAR 6 months after PPCI, given that their index value of left ventricular end-diastolic volume (LVEDV) increased 37.91 ± 14.31%, as compared with their values at the hospital discharge. We also observed a significant difference in the level of troponin-T (TnT) and creatine kinase (CK), but not in the elevation of the ST segment, or in the timing of infarction pain during patient admission. In addition, there were no significant differences between LVAR and non-LVAR groups in terms of gender, age, or cardiovascular risk factors.

**TABLE 1:** Demographic and clinical characteristics of STEMI patients (*n* = 38).

	STEMI patients (*n* = 38)	Non-LVAR (*n* = 26)	LVAR (*n* = 12)	*p*-value
Age (years)	58 ± 10	57 ± 10	60 ± 9	0.420
Male sex	35 (92.10%)	24 (92.30%)	11 (91.66%)	0.949
Arterial hypertension	14 (36.84%)	7 (26.92%)	7 (58.33%)	0.085
Smoking	18 (47.37%)	12 (46.15%)	6 (50.00%)	0.834
Dyslipidemia	18 (47.37%)	13 (50.00%)	5 (41.67%)	0.647
Type 2 diabetes mellitus	7 (18.42%)	6 (23.08%)	1 (8.33%)	0.288
LVEDV (ml/m^2^)^$$^	68.19 ± 20.63	65.99 ± 16.36	72.60 ± 27.62	0.453
LVEDV (ml/m^2^)^$$$^	75.84 ± 28.13	62.58 ± 14.85	102.38 ± 30.08	< 0.001*
Creatine kinase (mg/dL)^$^	2187 (1044–4611)	1590 (723.5–3335)	4056 (2165–5090)	0.028*
Troponin-T (ng/mL)^$^	5835 (3642–11493)	4785 (3503–6802)	11215 (4921–14129)	0.033*
ST elevation	13 (8.25–20.50)	11 (8.00–16.75)	18 (10.75–21.75)	0.1854
Timing of infarction pain	160 (120–230)	140 (120–235)	180 (150–250)	0.3810

Patients were classified into two groups: no remodeling (n = 26) and remodeling (n = 12). Data are shown as the means ± SEM or median (IQR) and as n (%). Student’s t-tests or Mann-Whitney test were performed. (*)Indicates significance at p < 0.05. (^$^)Indicates that the variable was examined at admission, ^$$^ at hospital discharge and ^$$$^ at 6 months after PPCI.

*IQR, interquartile range; LVAR, left ventricular adverse remodeling; LVEDV, left ventricular end-diastolic volume; PPCI, primary percutaneous coronary intervention; SEM, standard error of the mean; STEMI, myocardial infarction with ST-segment elevation. Significant p-values are provided in bold.*

**FIGURE 2:**
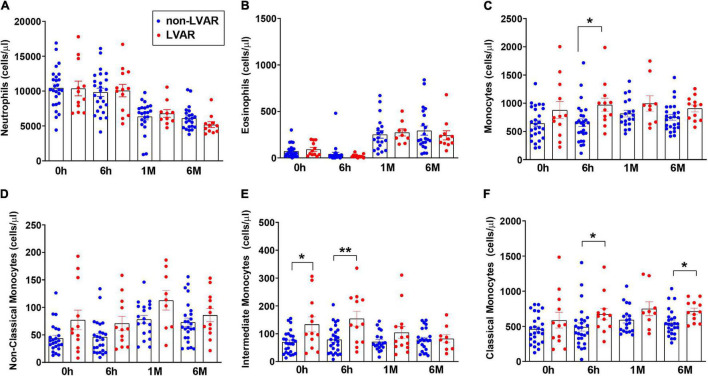
Analysis of the level of granulocytes, monocytes and monocytes subsets in patients with or without LVAR. **(A–F)** Bar graphs show the level of neutrophils **(A)**, eosinophils **(B)**, total monocytes **(C)** and the monocyte subsets: non-classical **(D)**, intermediate **(E)** and classical monocytes **(F)** in PB samples of non-LVAR STEMI patients (blue dots, *n* = 26) and STEMI patients who developed LVAR (red dots, *n* = 12) at 0, 6 h, 1 and 6 months post PPCI. Data are shown as the means ± SEM. Kruskal–Wallis test with multiple comparisons corrected by Dunn’s test was performed. (*) and (**) indicate significance at *p* < 0.05 and *p* < 0.01, respectively. LVAR: left ventricle adverse remodeling; PB, peripheral blood; PPCI, primary percutaneous coronary intervention; STEMI, ST-elevation myocardial infarction.

To characterize the relevance of the observed changes in the level of inflammatory cells, we examined whether they were different in patients who developed LVAR compared with those who did not present cardiovascular adverse events after PPCI. Figure 2 shows that there were no significant differences in the level of neutrophils (Figure 2A) and eosinophils (Figure 2B) between the two groups of patients. However, there was a significant difference in total monocyte counts 6 h after PPCI (Figure 2C). We further analyzed the level of all monocyte subset populations. Figure 2D shows that there was no difference in the level of non-classical monocytes at any time points analyzed. By contrast, levels of pro-inflammatory intermediate monocytes were increased significantly in patients who developed LVAR before and 6 h after PPCI (Figure 2E). Meanwhile, classical monocytes increased significantly 6 h after PPCI in patients with LVAR compared with those without LVAR (Figure 2F). Moreover, classical monocytes were still increased 6 months after the intervention. These data indicate that these two monocyte subset populations are likely associated with the appearance of the LVAR.

### Correlation of Pro-Inflammatory Intermediate Monocyte Levels Increase With Left Ventricular Adverse Remodeling

**FIGURE 3:**
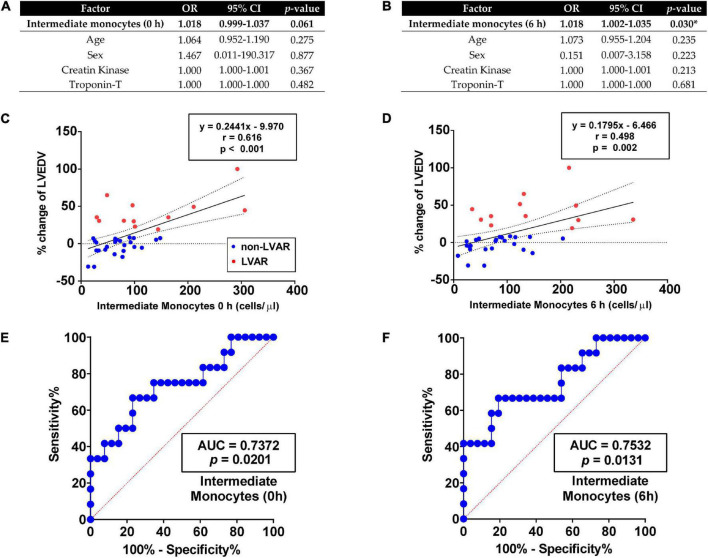
Correlation analysis of the level of intermediate monocytes with LVAR. **(A,B)** Multivariate logistic regressions analysis of intermediate monocytes measured in PB at 0 h **(A)** and 6 h **(B)**, using age, sex, creatine kinase and troponin-T as cofactors. **(C,D)** Linear regression curves analysis using the percentage of change in LVEDV of non-LVAR (blue dots, *n* = 26) and LVAR (red dots, *n* = 12) patients as dependent variable and intermediate monocytes level at 0 h **(C)** or 6 h **(D)** as independent factor. **(E,F)** ROC analysis with the AUC (values given on the graphs) indicating sensitivity and specificity of level of intermediate monocytes at 0 h **(E)** or 6 h **(F)** to predict LVAR. Multivariate logistic regression, linear regression and ROC analysis were performed. (*) Indicates significance at *p* < 0.05. AUC, area under the curve; CI, confidence interval; LVAR, left ventricle adverse remodeling; LVEDV, left ventricular end-diastolic volume; OR, odds ratio; PB, peripheral blood; ROC, Receiver-operating characteristics.

To distinguish which monocyte subset population could be a fair predictive marker for the appearance of LVAR in STEMI patients after PPCI we performed a multivariate logistic regression analysis. We calculated the odd ratio (OR) of different variables as age, sex, CK, and TnT levels, compared to the level of intermediate (Figures 3A,B) and classical (Supplementary Figures 2A,B) monocytes before and after revascularization. Multivariate analysis identified the level of intermediate monocytes as an independent predictor of LVAR, especially 6 h (*p* = 0.03) after revascularization (Figure 3B). However, multivariate analysis of classical monocytes suggests that they were not independent predictor of LVAR, since the OR was not significant (*p* > 0.05) at 0 or 6 h after the PPCI (Supplementary Figures 2A,B).

Linear regression analysis further confirmed that changes in the level of intermediate monocytes were associated with changes of the percentage of LVEDV, indicative of LVAR development. As illustrated in Figures 3C,D the levels of intermediate monocytes at 0 h (*r* = 0.616, *p* < 0.001), and 6 h after the PPCI (*r* = 0.498, *p* = 0.002) significantly correlated with changes in the percentage of the LVEDV. In addition, in Figures 3E,F, ROC curves analysis showed that the area under the curve (AUC) of these monocytes at 0 and 6 h, were higher than 0.7 with significant *p*-values, indicating an accurate level of adjustment. In contrast, the linear regression curves of classical monocytes presented a worse association with the changes in the percentage of the LVEDV with an “*r*” below 0.30 (Supplementary Figures 2C,D). Similarly, ROC curves with classical monocytes at 0 h showed poor sensitivity and specificity to predict LVAR (Supplementary Figure 2E). However, the AUC was higher than 0.7 at 6 h with *p* = 0.02 (Supplementary Figure 2F).

We also performed a ROC analysis with other classical parameters before and after revascularization, such as CK and troponin-T levels, time until revascularization, ST elevation in the emergency room and age. The AUC of CK and TnT levels at 0 h were higher than 0.70 with a significant *p*-value, although the multivariate analysis showed that these biomarkers were not independent of age and sex in predicting the LVAR (Figures 3A,B and Supplementary Figures 2A,B). The rest of the parameters provided weak AUC in the ROC analysis with insignificant *p*-values.

Taken together, these results confirmed that intermediate monocytes, rather than classical monocytes, are the best subset cell population to predict LVAR in STEMI patients after a PPCI.

### Analysis of Pro-inflammatory Cytokines in the Serum of ST-Segment Elevation Myocardial Infarction Patients

The main effectors of inflammation are cytokines, chemokines, and inflammatory mediators expressed and secreted from inflammatory cells in the tissues at the sites of infiltration. Therefore, we measured pro-inflammatory cytokines whose level could correlate with the appearance of LVAR. We performed a Bioplex analysis of 27 pro-inflammatory cytokines in the serum of a 13 patients’ subgroup (10 non-LVAR and 3 LVAR patients). We observed a significant increase in the secretion of Granulocyte-macrophage colony stimulating factor (GM-CSF) before PPCI (0 h), and interleukin 1β (IL-1β), interferon γ (IFNγ), interleukin 17 (IL-17), and vascular endothelial growth factor (VEGF) 6 h after PPCI in patients with LVAR (Supplementary Table 1). By contrast, there were no significant differences in other well-known pro-inflammatory 12 cytokines, as interleukin 1 receptor antagonist (IL-1Ra), tumor necrosis factor α (TNF-α), interleukin 4 (IL-4), interferon γ –inducible protein (IP-10), interleukin 9 (IL-9), interleukin 8 (IL-8), granulocyte colony stimulating factor (G-CSF), fibroblast growth factor basic (FGF basic), macrophage inflammatory protein 1 α (MIP-1α), macrophage inflammatory protein 1 β (MIP-1β), or platelet-derived growth factor BB, and Eotaxin. Next, we performed ELISA to measure the level of these differentially expressed cytokines at the time point of their maximum level of secretion (6 h). As illustrated in Supplementary Figures 4A–D, only VEGF increased differentially in LVAR patients, compared with non-LVAR patients. We also observed an incremental trend in the level of the IL-1β. Likewise, ROC curves showed that only the AUC of the levels of VEGF was higher than 0.7 to predict LVAR (Supplementary Figures 4E–H).

### Study of miRNAs Expressed by Inflammatory Cells in Patients With ST-Segment Elevation Myocardial Infarction

**FIGURE 4:**
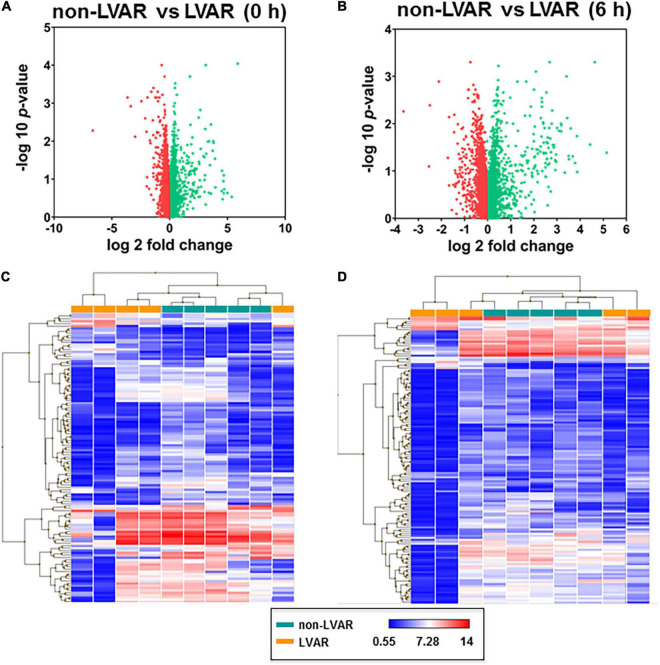
miRNAs differentially expressed between non-LVAR and LVAR patients in PBMCs. **(A,B)** Volcano plots showing miRNAs differentially expressed in LVAR patients compared to non-LVAR patients at 0 h **(A)** and 6 h **(B)** after PPCI (Green dots: up-regulated miRNAs, red dots: down-regulated miRNAs). **(C,D)** Hierarchical clustered sample-centric heat-map plots of –2.5 > fold change > 2.5 value of miRNAs in PBMCs samples from non-LVAR (blue rectangle, *n* = 5) and LVAR patients (orange rectangle, *n* = 5) at 0 h **(C)** and 6h **(D)**. Scale bar, downregulated (blue) and upregulated (red). LVAR, left ventricle adverse remodeling; miRNAs, microRNAs; PBMCs, peripheral blood mononuclear cells; PPCI, primary percutaneous coronary intervention.

Next, we analyzed the expression of miRNAs in PBMCs isolated from the control healthy group (*n* = 3), a group of non-LVAR (*n* = 5), and LVAR STEMI patients (*n* = 5), before (0 h) and after revascularization (6 h). We found only 256 miRNAs differentially expressed in control compared with the non-LVAR patients (fold change ± 2.5 and *p* < 0.05); meanwhile, 925 miRNAs were differentially expressed between control and LVAR patients (data not shown). Interestingly, we found 577 and 1,209 miRNAs differentially expressed in PBMCs from LVAR, as compared with the non-LVAR patients before and after revascularization, respectively (Figures 4A,B). Volcano plots show that 154 miRNAs were downregulated and 423 were upregulated (Figure 4A) before PPCI, and 85 miRNAs were downregulated and 1,124 were upregulated (Figure 4B) 6 h after revascularization, in LVAR patients compared with the non-LVAR patients (fold change ± 2.5 and *p* < 0.05). The hierarchical clustering analysis confirmed that miRNAs were differentially expressed in the two groups of patients with STEMI and they were well clustered (Figures 4C,D).

**FIGURE 5:**
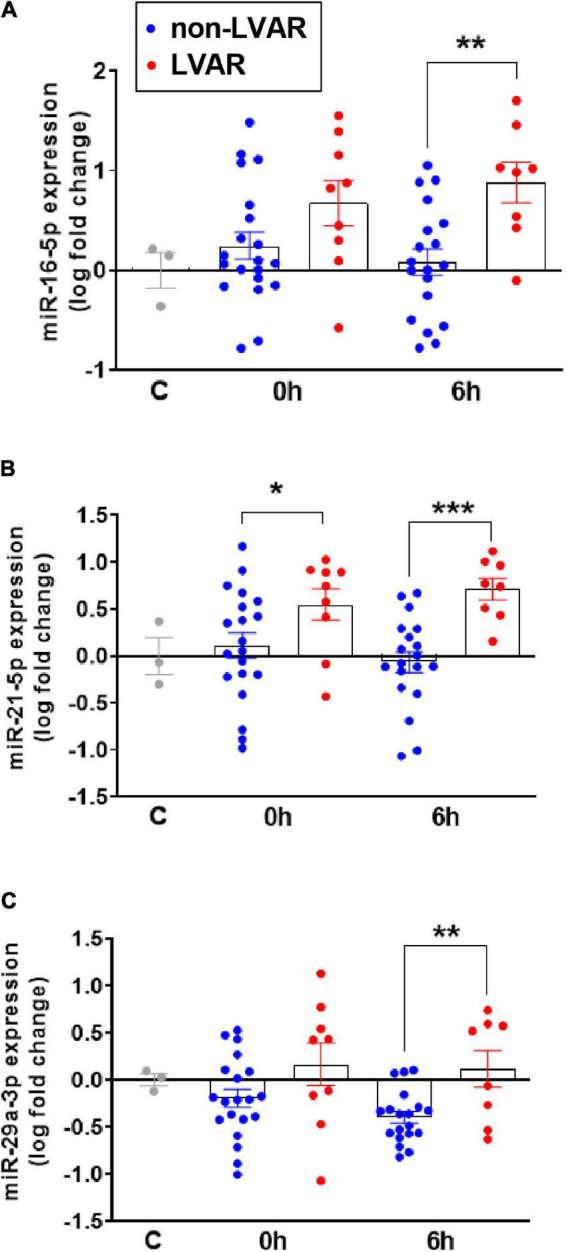
Analysis of miR-16, miR-21 and miR-29a expression in PBMCs from non-LVAR and LVAR patients. **(A–C)** Bar graphs show the levels of expression of miR-16 **(A)**, miR-21 **(B)** and miR-29a **(C)** in controls and non-LVAR (blue dots, *n* = 20) and LVAR patients (red dots, *n* = 9), pre-PPCI (0 h) and post-PPCI (6 h). Values are presented as the means ± SEM. Ordinary one-way ANOVA with multiples comparisons using *t*-test without correction (Fisher’s LSD test) was performed. (*) indicates *p* < 0.05, (**) indicates *p* < 0.01 and (***) indicates *p* < 0.001. LVAR, left ventricle adverse remodeling; PBMCs, peripheral blood mononuclear cells; PPCI, primary percutaneous coronary intervention.

Based on these findings and using *in silico* databases, we selected miRNAs suggested to target cytokine production (Supplementary Table 2), focusing on those that might regulate the oversecreted cytokines in LVAR patients, 6 h after PPCI detected in Bioplex analysis. Therefore, we chose 3 miRNAs, miR-16-5p, miR-21-5p, and miR-29a-3p that are predicted to target IL-1β, IFNγ, and VEGF genes. We did not find any miRNA, from the microarray list, targeting IL-17 gene, possibly because of that this cytokine is expressed and secreted mainly by lymphocytes (14). Using qRT-PCR we confirmed significant increase in the level of miR-16-5p, miR-21-5p, and miR-29a-3p in LVAR vs. non-LVAR patients, especially 6 h after revascularization (Figures 5A–C). ROC curves analysis indicated that the AUC of miR-16-5p, miR-21-5p, and miR-29a-3p were statistically significant 6 h after PPCI (Supplementary Figure 5). Using the online platform Panther to analyze potential target genes of these miRNAs, we found a set of genes involved in immune response and inflammation mediated by cytokine, IFNγ, and IL signaling pathways (detail in Supplementary Table 3), which support our hypothesis.

### Analysis of Combined Biomarkers to Improve Prediction of Left Ventricular Adverse Remodeling

**FIGURE 6:**
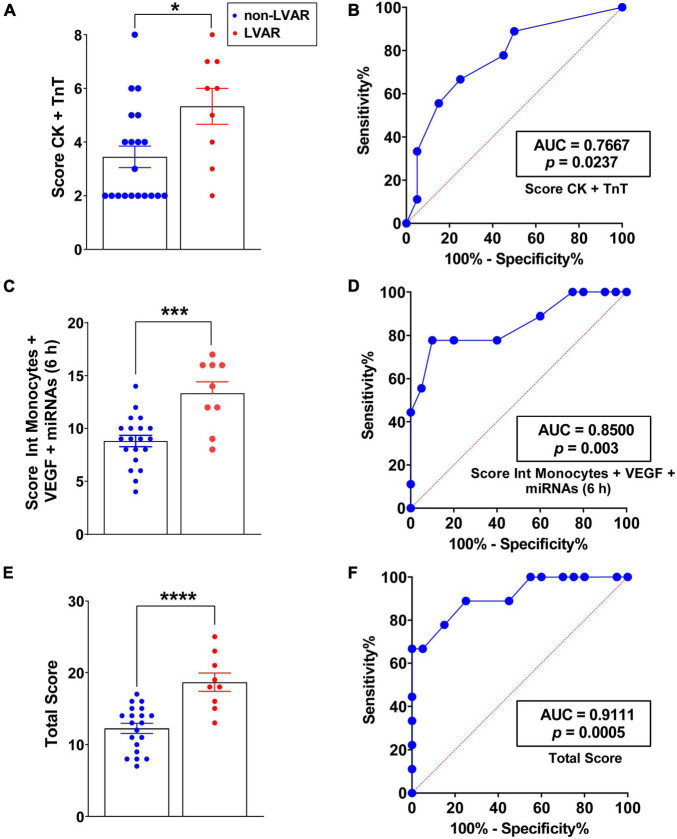
Analysis of the level of the remodeling prediction score in patients with or without LVAR. **(A)** Bar graphs show the CK and TnT, which is the sum of the score, from 1 to 4, assigned to each level of CK and TnT at 0 h, in non-LVAR (blue dots, *n* = 20) and LVAR (red dots, *n* = 9) patients (see section Materials and Methods” and Supplementary Table 3 for more information about formula of the scores and the ranges). **(B)** ROC analysis with the AUC (values given on the graphs), indicating sensitivity and specificity of the CK and TnT score **(A)** at 0 h to predict LVAR. **(C)** Bar graphs show intermediate monocytes, VEGF and miRNAs score at 6 h in non-LVAR and LVAR patients. **(D)** ROC analysis of intermediate monocytes, VEGF and miRNAs score at 6 h **(C)** to predict LVAR. **(E)** Bar graph shows the total score (summary of assigned score to CK and TnT at 0 h, and intermediate monocytes, VEGF and miRNAs at 6 h post-PPCI) in non-LVAR and LVAR patients. **(F)** ROC analysis with the AUC (values given on the graphs), indicating sensitivity and specificity of the total score to predict LVAR. Values are presented as the means ± SEM. Mann-Whitney (non-parametric) and *t*-test (parametric) and ROC analysis were performed. (*), (***), and (****) indicate significance at *p* < 0.05, *p* < 0.001 and *p* < 0.0001, respectively. AUC, area under the curve; CK, creatine kinase; LVAR, left ventricle adverse remodeling; miRNAs, microRNAs; PPCI, primary percutaneous coronary intervention; ROC, Receiver-operating characteristics; TnT, troponin-T; VEGF, vascular endothelial growth factor.

Looking for better markers to more accurately identify patients who will develop LVAR, we performed combined ROC analysis using classical markers, such as CK and TnT levels at 0 h, and newly identified markers, intermediate monocytes, VEGF, and miRNAs levels at 6 h after PPCI. As described earlier, the analysis of each factor independently showed an AUC greater than 0.70 with significant *p* values. To improve the power of LVAR prediction, we established a worsening diagnosis score based on the secretion level of all biomarkers in each patient as described in Supplementary Table 4 and Supplementary Materials and Methods. First, we calculated the score by combining the analysis of classical parameters CK and TnT. As depicted in Figure 6A, LVAR patients obtained a significantly higher score compared with the non-LVAR patients. Figure 6B indicates that the analysis of the ROC curve of this score was more accurate in predicting LVAR, than those of CK and TnT analyzed separately (Supplementary Figures 3A,C). Next, we compared the predictor score combining the analysis of the new markers, pro-inflammatory intermediate monocytes, VEGF, miR-16-5p, miR-21-5p, and miR-29a-3p, 6 h after revascularization. Figures 6C,D shows that significance was even higher for LVAR patients compared with non-LVAR patients, and ROC curve analysis presented an AUC of 0.85 with a *p*-value of 0.003. Finally, as shown in Figures 6E,F, the combined analysis of the score of all the examined markers, CK, TnT, pro-inflammatory intermediate monocytes, VEGF, miR-16-5p, miR-21-5p, and miR-29a-3p provided a significantly higher score and the ROC curve achieved a great AUC of 0.9111 with a *p* = 0.0005 (Figure 6F), to predict LVAR appearance in STEMI patients after an PPCI. Therefore, this data analysis suggests that a patient with a score higher than 14.50 (being 25 the maximum score) will positively develop LVAR, with 75.00% of sensitivity and 88.89% of specificity, as indicated in Figure 6F. These findings revealed that the combined analysis of classical and new biomarkers (CK, TnT, VEGF, intermediate monocytes, and miRNA levels) provides highly specific and sensitive value to predict LVAR in patients undergoing PPCI.

## Discussion

Acute myocardial infarction and the consequent HF are major causes of mortality and morbidity worldwide. There is a general consensus that early and prompt coronary intervention has significantly improved outcomes in the acute phase of AMI. However, there is still a great incidence of LVAR in STEMI patients who underwent successful PPCI. Patients enrolled in this study were admitted at the hospital less than 12 h after the onset of chest pain and without having previous history of ischemic heart disease, showing a TIMI (Thrombolysis in Myocardial Infarction) flow of 0 in the left anterior descendent coronary artery. The inclusion criteria were rigorous to guarantee consistent results related to the impact of revascularization on inflammatory cells measurements, and the release of cytokines and miRNAs. Almost 32% of patients with STEMI developed LVAR as soon as 6 months after PPCI in our cohort, which agree with other finding (15). In this study, we found that the classical cardiac markers, CK and TnT, were significantly higher in patients who developed LVAR, compared with those without LVAR, which is concordant with a large infarct size of the heart. However, multivariate statistical analysis using these classical markers indicated that they are weak predictors of LVAR after PPCI.

Compelling evidences highlighted the role of the inflammatory response to AMI and its critical role in determining the infarct size and subsequent LVAR (2). However, the use of anti-inflammatory drugs has not achieved any substantial benefits in the clinical trials (16). Here, we focused on the role of monocytes as central regulators of inflammatory process after revascularization (5). Our data revealed that the levels of classical and intermediate monocytes were significantly increased in STEMI patients who later developed LVAR after an PPCI. Previous studies demonstrated significant increase in monocyte subsets levels in non-revascularized STEMI patients (4, 9) and in non-STEMI patients (17, 18). They also suggested that the level of classical monocytes correlated with a reduced left ventricle ejection fraction (LVEF) and a larger infarct size (17, 18). Interestingly, according to our linear regression analysis, the increase in the level of intermediate monocytes was associated significantly with changes in the LVEDV, and their levels are well-clustered in non-LVAR and LVAR patients. Moreover, multivariate regression and ROC analysis indicated that changes in the level of these monocytes were associated with LVAR appearance and provided very accurate sensitivity and specificity. These data also showed that this monocyte subset, but not CK, neither TnT nor sex, nor age, could reliably predict the appearance of LVAR. At the same time, our data demonstrated that classical monocytes could not be considered as an independent predictor of LVAR. Recently, some studies used the level ratio between monocytes and lymphocytes as a prognostic marker for non-STEMI patients (19), or with eosinophils for STEMI patients (20), and with platelets (21) in AMI patients. However, none of these studies analyzed the correlation between monocyte subsets with LVAR. Actually, the importance of all those inflammatory cells populations and their roles after an ischemic event constitute an interesting research field, even if they are still under debate (22).

The inflammatory response is characterized by cytokines and chemokines secretion which can determine the final recovery of ischemic tissue and the occurrence of the LVAR (7). Very few studies analyzed the level of multiple cytokines in STEMI patients after PPCI. In our study, within 27 pro-inflammatory cytokines, we found a significant increase in the level of VEGF in patients who developed LVAR 6 h after PPCI. Consistently, other studies highlighted the role of VEGF isoforms to predict MACEs in the clinical practice (23); in addition, the plasma increased level of VEGF was associated with microvascular obstructions and poor prognosis in STEMI patients as described recently in the PREGICA study (ClinicalTrials.gov identifier: NCT01113268) (24). Therefore, a substantial increase in the level of VEGF after PPCI might be detrimental for heart recovery from the ischemic events. In addition to inflammatory cells subsets and cytokines, we also studied the behavior of miRNAs in STEMI patients. Recently, we provided evidence indicating that PBMCs release miRNAs into the blood stream of patients with LVAR (12). In this study, we observed significant dysregulation of miRNAs levels in PBMCs owing to patients who later developed LVAR compared with those without LVAR. Based on miRNAs array and *in silico* analysis we found that three miRNAs, miR-16-5p, miR-21-5p, and miR-29a-3p, are predicted to target genes involved in the inflammatory signaling pathway. miR-21 is a well-known miRNA involved in cardiovascular diseases, as reviewed recently (25). Several reports described changes in the expression of miR-21 in cardiomyocytes, fibroblast, and endothelial cells during ischemic heart disease, but little is known regarding its possible secretion by inflammatory cells after an AMI. Interestingly and in agreement with our finding, exosomal miR-21 is secreted from macrophages, influencing the migration and proliferation of smooth vascular cells in the atheroma plaque (26). On the other hand, miR-16-5p and miR-29a-3p seem to participate in inflammation process in atherosclerosis (27, 28). Although, the role of miR16-5p and the inflammatory process in heart has been barley addressed. Meanwhile, miR-29 family was associated with LVAR after myocardial infarction in the mouse model (29). Another study determined that changes in the expression of miR-29a-3p was associated with sudden death in patients with coronary heart disease, apparently originated from M1 polarized pro-inflammatory monocytes, correlating with the increase in the level of pro-inflammatory cytokine IL-6 (30). Recently, we demonstrated that the overexpression of miR-29a-3p prevents changes in the expression of apoptotic and fibrotic genes induced by ischemia and reperfusion in rats (31).

Interestingly, our data demonstrated that those three miRNAs and their predicted target cytokines are upregulated in PBMCs and serum, respectively, in patients with LVAR, compared with non-LVAR patients, at the same time point, 6 h after patients’ intervention. Based on these data we cannot assure whether miRNAs are up or downstream cytokines production. However, we do not discard that under these conditions, inflammatory cells may increase miRNAs expression in a way to control their own exacerbated expression of cytokines. This intriguing bidirectional miRNA-cytokine regulation was already described in other inflammatory cells, such as primary helper T cells, which were suggested to increase miR-29 expression to correct their aberrant expression of IFNγ (32). In agreement with this analysis, independent studies also showed bidirectional regulation between cytokines and miRNAs (33, 34).

Since it is difficult to predict the outcome of a such complex and multifactorial disease as AMI, we have combined the analysis of intermediate monocytes, VEGF, and miRNAs using a score to stratify STEMI patient and to assess their value as powerful prognostic biomarkers for LVAR development. In fact, the combined analysis measuring the levels of intermediate monocytes, the release of VEGF, and miRNAs provided greater statistical significance compared to the analysis of only classical markers. We demonstrate that combined analysis of classical markers CK and TnT generated an AUC of 0.7667 (*p* = 0.0237), while the combined analysis of intermediate monocytes, VEGF, miR-16, miR-21, and miR-29a generated an AUC of 0.8500 (*p* = 0.003). Interestingly, the joint analysis of classical markers together with the new identified markers analyzed in this study achieved an AUC of 0.911 with a *p* = 0.0005. These findings ensure a great precision to predict LVAR in revascularized patients using this combined analysis. In accordance with our results, a recent study demonstrated that combined analysis of 8 miRNAs and NT-proBNP provided a potent diagnosis results for HF detection (35).

In conclusion, the analysis of combined parameters such as CK, TnT, intermediate monocytes, VEGF, and miRNAs levels, could be useful for an early identification of patients who will develop LVAR. Herein, we demonstrate that the increase in the level of intermediate monocytes, VEGF, and miRNAs in the bloodstream, at the moment of the ischemic event, can serve as more sensitive predictive markers, rather than simple blood indexes, since their combined analysis accurately predicted whose patients have high probability to develop an LVAR and HF in future. Therefore, prophylactic therapy in these patients could prevent HF. Further studies are eagerly needed to establish the therapeutic effect of targeting inflammatory cells subset, miRNAs, and cytokines in STEMI patients undergoing PPCI.

## Data Availability Statement

The datasets presented in this study can be found in online repositories. The names of the repository/repositories and accession number(s) can be found below: ArrayExpress Browser, E-MTAB-11002.

## Ethics Statement

The studies involving human participants were reviewed and approved by the Local Ethics Committee on Human Research at the University Hospital “Virgen del Rocio” of Seville (Approval no. 2013PI/096). The patients/participants provided their written informed consent to participate in this study.

## Author Contributions

IG-O, RDT, EB, FG-M, DF, AG-R, LD-L, GB-E, AO-F, and TS: conceptualization, data acquisition, analysis, and interpretation. IG-O, RDT, EB, FG-M, GB-E, and TS: investigation and methodology. RDT, AO-F, and TS: writing original draft. AG-R, FG-M, LD-L, GB-E, and AO-F: revising the draft and clinical concepts. AO-F and TS: funding acquisition project administration. All authors contributed to the article and approved the submitted version.

## Conflict of Interest

The authors declare that the research was conducted in the absence of any commercial or financial relationships that could be construed as a potential conflict of interest.

## Publisher’s Note

All claims expressed in this article are solely those of the authors and do not necessarily represent those of their affiliated organizations, or those of the publisher, the editors and the reviewers. Any product that may be evaluated in this article, or claim that may be made by its manufacturer, is not guaranteed or endorsed by the publisher.
