# Supplementary material for: Predicted Value of MicroRNAs, Vascular Endothelial Growth Factor, and Intermediate Monocytes in the Left Adverse Ventricular Remodeling in Revascularized ST-Segment Elevation Myocardial Infarction Patients
Source: Front Cardiovasc Med. 2022 Mar 24;9:777717. doi: 10.3389/fcvm.2022.777717 (PMC8987717; doi:10.3389/fcvm.2022.777717)
Supplement: Supplementary file 1 [file Presentation_1.pdf]

Supplementary Material

1 Supplementary Data

1. Study Subject

All the subjects of this study signed an informed consent form and are voluntary participants. As shown in the STROBE (Supplemental Figure 1), a cohort of 72 patients, were prospectively recruited between March 11th 2016 and February 10th 2020. The participants were divided into two groups: 28 healthy volunteers without diagnosed coronary disease and 44 who underwent coronary artery angiography at the University Hospital "Virgen del Rocio" of Seville and were diagnosed with STEMI and treated with PPCI. Clinical and demographical information were collected at the admission in the hospital and during each follow-up visit, scheduled at 1 and 6 months after the hospital discharge. In the STEMI group 4 patients (9.09%) died from adverse cardiovascular events, 2 missed the follow-up, and 38 (86.36%) completed the 6-months follow-up until September 2020.

The inclusion criteria were: age less than 75 years; patients with STEMI due to occlusion of the left descending artery with an epicardial blood flow TIMI (Thrombolysis in Myocardial Infarction) grade 0-1 in the initial angiogram treated with PPCI, with the onset of symptoms less than 12 hours before the angioplasty. Patients with ischemic heart disease history, a <30 ml/min glomerular filtration rate, and a TIMI flow grade over 1 were excluded.

All the patients were selected for a primary percutaneous coronary intervention through the right radial artery (100% of the STEMI patients). The procedure was performed, at the beginning of the intervention, with the administration of a heparin bolus of 10,000 units. Judkins large-lumen guiding catheter and guidewire were used. Coronary arteries were visualized using angiogram. Before accessing through the radial artery, palmar arch good circulation was confirmed to avoid hand ischemia. After that, a manual thrombus aspiration was systematically performed. To implant the stent the guidewire was appropriately positioned in the stenotic segment of the coronary artery; the balloon was later expanded and the stent was delivered in the corresponding position, relieving the stenosis. All patients in this study were treated with drug-eluting coronary stents with antiproliferative effects. After the intervention all the patients had a TIMI 3, meaning totally blood flow recovery. During the period at the hospital and before patient discharge, 100% of the patients were treated with an oral anticoagulant (aspirin) for 2 months, and antiaggregant (clopidrogel) for 6 months

2. Echocardiographic studies

Before patient discharge (72 hours post-STEMI) and 6 months after PPCI procedure, an echocardiography was completed to measure left ventricular ejection fraction (EF) systolic and diastolic left ventricular diameters and volumes in order to evaluate the development of left ventricular adverse remodelling (LVAR). A non-invasive ultrasound scan was performed to analyze cardiac volumes and function. The study was carried out with an iE33 (Philips, Amsterdam, Netherlands) echo system with images recorded in DICOM format at the Xcelera station for analysis. Dynamic sequences of three consecutive heartbeats were acquired with image optimization in 2D and doppler-color in several views: pure long axis parasternal, short axis parasternal at mitral valve level, papillary muscles and apex. Moreover, plane apical 2, 3, 4 and 5 chambers views were acquired to visualize segmental

contractility defects and to calculate left ventricle ejection fraction, and end-diastolic and end-systolic left ventricle volume (Simpson 4C) (1). Subcostal view was acquired to visualize the inferior vena cava and the pericardial effusion. Continuous or pulsed Doppler (as appropriate) at aortic valve level and left ventricular outflow tract (LVOT), mitral filling, pulmonary veins, tricuspid filling and pulmonary valve were routinely evaluated. In case of valve insufficiency, appropriate measurements were made as established in valvulopathies clinical practice guidelines(2). Pulse Doppler and tissue Doppler in mitral septal and lateral ring were measured to estimate diastolic function and end-diastolic left ventricle pressure. Tricuspid annular systolic displacement (TAPSE) was used to calculate right ventricular systolic function, and tricuspid regurgitation was used to estimate pulmonary artery pressure.

Patients with an increase > 20% in left ventricular end-diastolic volume (LVEDV) and a left ventricular ejection fraction less than 50% were considered as LVAR patients, according to current clinical indications (3,4).

3. Blood samples: extraction and preparation

Before initiation of the PPCI procedure, patients' blood samples were extracted after catheter insertion in the radial or femoral arteries. These samples represented the time point before revascularization (0h). Moreover, we collected additional blood samples at 6-12 h after culprit vessel opening at 1 and 6 months after the ischemic event. We obtained serum from the blood samples collected without antiserum (in BD Vacutainer ® SST ™ II Advance tubes; Becton, Dickinson and Company, NJ, USA) using centrifugation at 2500 rpm at 4°C for 15 minutes. Fresh peripheral blood samples were collected into ethylenediaminetetraacetic acid (EDTA)-coated tubes (BD Vacutainer® K2E; Becton, Dickinson and Company, NJ, USA) and processed within 6h post- collection. From these tubes, 100 µl of blood were incubated with 10 µl of CD11b (APC conjugated, BD Pharmingen™ APC Mouse Anti-Human CD11b/Mac-1 clon ICRF; BD Pharmingen, CA, USA), 10 µl of CD14 (FITC conjugated, BD Pharmingen™ FITC Mouse Anti-Human CD14 clon M5E2; BD Pharmingen, CA, USA), 10 µl of CD16 (PE conjugated, BD Pharmingen™ PE Mouse Anti-Human CD16 clon 3G8; BD Pharmingen, CA, USA) and 2.5 µl of CD66b (PerCP-Cy5 Tandem dye conjugated, BD Pharmingen™ PerCP-Cy™5.5 Mouse Anti-Human CD66b clon G10F5; BD Pharmingen, CA, USA) during 30 minutes at room temperature (RT) protected from the light. Red blood cells were then lysed with 2 ml of a 10-fold dilution of BD FACS™ Lysing Solution 10X Concentrate (BD Biosciences, NJ, USA) with distilled water. After 10 min at RT, the resulting cell suspension was washed with phosphate-buffered saline (PBS 1X; GIBCO, Paisley, UK) and centrifuged at 1800 rpm during 8 min. The pellet was resuspended in 300 µl of PBS 1X and then analysed with flow cytometry.

4. Flow Cytometry

Blood samples were acquired immediately after the staining protocol in a Canto II flow cytometer (BD Biosciences, NJ, USA) with three lasers (405 nm 30mW, 488 nm 20mW, 640 nm 17 mW). The FACSDiva software 8.0 (BD Biosciences, NJ, USA) was used for sample acquisitions and analysis. We analysed the inflammatory cell populations: neutrophils (CD16++CD66b+), eosinophils (CD16+CD66b-) and monocyte subsets. Monocyte subgroups were classified as CD14++/CD16– (classical), CD14++/CD16+ (intermediate), and CD14+ /CD16++ (non-classical) and absolute counts (expressed as number of cells/µl) were calculated by multiplying the percentage of each subset by the leucocyte counts of each patient.

5. Study of the cytokine profile in patients' serum

We evaluated the level of 27 cytokines (FGF basic, Eotaxina, G-CSF, GM-CSF, IFN-γ, IL-1 β, IL-1ra, IL-2, IL-4, IL-5, IL-6, IL-7, IL-8, IL-9, IL-10, IL-12, IL-13, IL-15, IL-17, IP-10, MCP-1, MIP-1α, MIP-1β, PDGF-BB, RANTES, TNF-α, VEGF) in serum using the Bio-Plex Pro™ Human Cytokine 27-plex Assay kit (Bio-Rad, CA, USA) following the manufacturer's instructions. Briefly, we added 40 µl of patients' serum diluted in 120 µl of sample diluent included in the kit into a 96-well plate. The well washing was made by the 10-fold dilution of 10X Magnetic Beads Bio-Plex™ (Bio-Rad, CA, USA) and the magnetic holder Bio-Plex Pro™ washstation (Bio-Rad, CA, USA). Finally, the 96-well fluorescence was measured on the Bio-Plex® 200 System (Bio-Rad, CA, USA) and analysed with the software Bio-Plex Systems 100 (Bio-Rad, CA, USA).

Next, we checked the levels of IFN-γ, IL-1β, VEGF and IL-17A in patients' serum using ELISA kits. Thus, levels of IFN-γ were measured using the solid-phase sandwich IFN-γ Human ELISA Kit (BMS228; ThermoFisher Scientific,Waltham, MA, US) according to the manufacturer's protocol. The assay range was 1.6-100 pg/mL. The minimal detectable concentration was 0.99 pg/mL with an intra-assay coefficient of variation (CV) of 4.5% and interassay 5.7%. Serum levels of IL-1β were detected using the solid-phase sandwich IL-1β Human ELISA Kit (BMS224-2; ThermoFisher Scientific, Waltham, MA, US) according to the manufacturer's protocol. The assay range was 3.9-250 pg/mL. The minimal detectable concentration was 0.3 pg/mL with an intra- assay CV of 5.1% and interassay 8.6%. Next, levels of VEGF were measured using the solid-phase sandwich VEGF-A Human ELISA Kit (BMS277-2; ThermoFisher Scientific, Waltham, MA, US) according to the manufacturer's protocol. The assay range was 15.6-1000 pg/mL. The minimal detectable concentration was 7.9 pg/mL with an CV of 6.2% and interassay 4.3%. Finally, serum levels of IL-17 were detected using the solid-phase sandwich IL-17A Human ELISA Kit (BMS2017; ThermoFisher Scientific, Waltham, MA, US) according to the manufacturer's protocol. The assay range was 1.6-100 pg/mL.The minimal detectable concentration was 0.5 pg/mL with an intra- assay CV) of 7.1% and interassay 9.1%. All ELISAs were done in 96-well plate and were measured on the microplate reader CLARIOstar Plus (BMG LabtechOrtenberg, Germany).

6. RNA extraction and quantification

RNA extraction was done using mirVana™ miRNA Isolation Kit (Life Technologies, Grand Island, NY, US) to extract small RNAs from peripheral blood mononuclear cells (PBMCs) following manufacturer's instruction. Briefly, after a low-speed centrifugation, PBMCs were washed with PBS 1X and lysed in Lysis/Binding solution supplied with the kit. Then, Acid-Phenol:Chloroform (ThermoFisher Scientific, Waltham, MA, US) were added to the lysate. After purification steps, RNA were eluted in 60 µl of RNase-free water. Finally, PBMCs' RNA was measured using Qubit miRNA assay kit (ThermoFisher Scientific, Waltham, MA, US) and fluorometric quantification (Qubit™ Scientific; ThermoFisher Scientific, Waltham, MA, US).

7. miRNA Arrays Analysis

The total RNA was labeled using the FlashTag® Biotin HSR labeling Kit (Thermo Fisher Scientific, Inc.) following instructions supplied in the user manual, and we used GeneChip® miRNA 4.0 arrays (ThermoFisher Scientific, Waltham, MA, US) to analysis miRNAs. Washing, staining (GeneChip® Fluidics Station 450; ThermoFisher Scientific, Waltham, MA, US), and scanning (GeneChip® Scanner 3000; ThermoFisher Scientific, Waltham, MA, US) were done following manufacturer's protocol. Briefly, importing CEL file, the analysis of miRNA level RMA+DABG-All

and the exporting of the results were done using Transcriptome Analysis Console (TAC) 4.0 software (ThermoFisher Scientific, Waltham, MA, US). A comparative analysis between LVAR patients, non-LVAR patients and controls samples was carried out using fold-change of over ±2.5 and an ANOVA analysis with a p-value < 0.05. Hierarchical clustering was performed using complete linkage and Euclidean distance as a measure of similarity for the differentially expressed with TAC 4.0 software.

8. RT-Real time PCR analysis (miRNA expression assay)

We obtained cDNA from 1 µg of small RNAs using miScript II RT Kit (QIAGEN, Hilden, Germany) and cDNA product was diluted up to 1:10. qPCR was performed in a FrameStar 384 Well PCR Plate (4titude, BIOKé, Leiden, the Netherlands). PCR mix included 10X universal primer (included in miScript SYBR Green PCR Kit; QIAGEN, Hilden, Germany), SYBR Green reactive (iTaq™ Universal SYBR Green Supermix; Biorad, CA, USA) and specific oligos for each miRNAs: miScript Primer Assay Hs_miR_16_2 (5'-UAGCAGCACGUAAAUAUUGGCG-3'), Hs_miR-21-5p (5'-UAGCUUAUCAGACUGAUGUUGA-3') and Hs_miR_29a-3p (5'-UAGCACCAUCUGAAAUCGGUUA-3') (QIAGEN, Hilden, Germany), and Hs_Snord95 (QIAGEN, Hilden, Germany). qRT-PCR were performed on the Applied Biosystems Viia7 Real-Time PCR System (ThermoFisher Scientific, Waltham, MA, US). The thermal cycling conditions were as follows: 95°C for 20s followed by 45 cycles of 95°C for 1 s and 60°C for 20s. Expression data were calculated like Log of Fold change in logarithmic scale obtained with the comparative cycle threshold CT (ΔΔCT) method, using Hs_Snord95 as endogenous control.

9. In silico targeted gene searching

In order to identify miRNAs targets gene of inflammatory pathways and biological processes, we performed an analysis using the Gene Ontology (GO) browser PANTHER (Protein Analysis THrough Evolutionary Relationships, Version 15.0, http://pantherdb.org/genelistanalysis.do).

10. Statistical analysis

Statical analyses were performed using SPSS (SPSS Inc. version 25.0 IBM, Armonk, NY, USA) and GraphPad (GraphPad Software Inc., San Diego, CA, USA). Data are expressed as means ± standard error of the means (SEM). We removed the outliers and determined the normality of the distributions using GraphPad. For Gaussian distributions, we did an ordinary One-way ANOVA where we compared all the groups between them (Fisher's LSD test). For nonparametric distributions, we used the Kruskal–Wallis test with multiple comparisons corrected by Dunn's test. Multivariate regression analysis was conducted using SPSS. LVAR was selected as the binary dependent variable and creatine kinase (CK), troponin-T, sex, age, and classical and intermediate monocytes as the covariates for the adjustment. Linear regression analysis was performed using the level of monocytes and the percentage of change in LVEDV as independent and dependent variable, respectively.

In order to categorize a multi panel for LVAR detection, we stratified STEMI patients according to the level of intermediate monocytes, four cytokines and the expression of miRNAs. The level of each biomarker was divided in 4 ranges and each range had a score value from 1 to 4 assigned. The

total score was made by the sum of the score reached by each biomarker, (see below algorithm and Supplemental Table 3). In this categorization, the linear combination of intermediate monocytes levels (in cells/□l), the level of secretion for cytokines (in pg/ml) and $\log_{10}$ fold change values for miRNAs were used to calculate the prediction score, where the higher the score, the higher the likelihood for the patient to develop LVAR. To evaluate the specificity and accuracy of the different biomarkers to predict the appearance of LVAR in revascularized STEMI patients, we used the receiver operating characteristic (ROC) curve.

11. Score algorithm

$$Total\ Score = \sum (Score\ CK + Score\ TnT + Score\ int\ monocytes + Score\ VEGF + Score\ miRNAs)$$

$$Score\ CK = Score\ Creatine\ Kinase\ (0h)$$

$$Score\ TnT = Score\ Troponin - T\ (0h)$$

$$Score\ int\ monocytes = Score\ Intermediate\ Monocytes$$

$$Score\ VEGF = Score\ VEGF$$

$$Score\ miRNAs = \sum (Score\ miR16 + Score\ miRNA21 + Score\ miR29)$$

$$k = \frac{Maximum - Minimum}{4}$$

$$Score = 1 \rightarrow Range\ 1 \rightarrow Min \leq x\ \leq Min + k$$

$$Score = 2 \rightarrow Range\ 2 \rightarrow Min + k \leq x\ \leq Min + 2k$$

$$Score = 3 \rightarrow Range\ 3 \rightarrow Min + 2k \leq x\ \leq Min + 3k$$

$$Score = 4 \rightarrow Range\ 4 \rightarrow Min + 3k \leq x\ \leq Max$$

References

1. Chengode S. Left ventricular global systolic function assessment by echocardiography. *Ann Card Anaesth* (2016) **19**:S26–S34. doi:10.4103/0971-9784.192617

2. Baumgartner H, Falk V, Bax JJ, De Bonis M, Hamm C, Johan Holm P, Iung B, Lancellotti P, Lansac E, Rodriguez Mu D, et al. 2017 ESC/EACTS Guidelines for the management of valvular heart disease The Task Force for the Management of Valvular Heart Disease of the European Society of Cardiology (ESC) and the European Association for Cardio-Thoracic

Surgery (EACTS) Document Review. *Thierry Folliguet* **1**: doi:10.1093/eurheartj/ehx391

3. Mannaerts HFJ, Van Der Heide JA, Kamp O, Stoel MG, Twisk J, Visser CA. Early identification of left ventricular remodelling after myocardial infarction, assessed by transthoracic 3D echocardiography. *Eur Heart J* (2004) **25**:680–687. doi:10.1016/j.ehj.2004.02.030

4. Lang RM, Bierig M, Devereux RB, Flachskampf FA, Foster E, Pellikka PA, Picard MH, Roman MJ, Seward J, Shanewise JS, et al. doi:10.1016/j.echo.2005.10.005. (2005) doi:10.1016/j.echo.2005.10.005

2 Supplementary Figures and Tables

2.1 Supplementary Figures

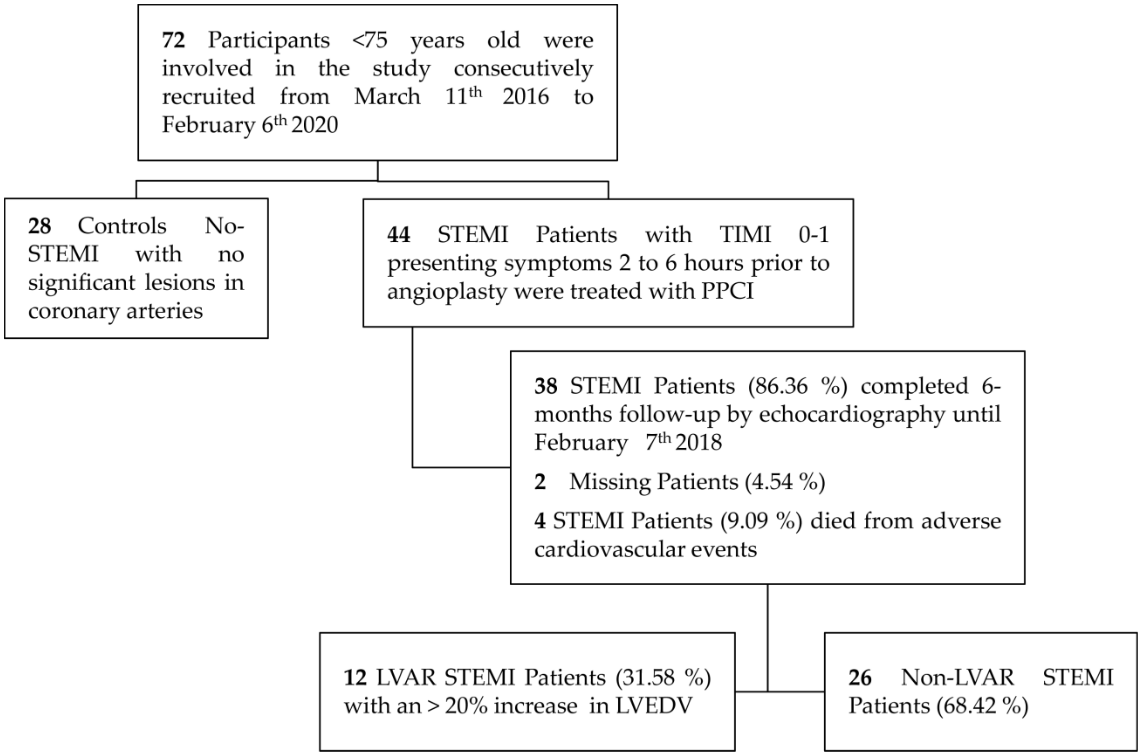

Supplemental Figure 1. STROBE diagram of the subjects and the study design. LVAR: Left ventricular adverse remodelling; LVEDV: left ventricular end-diastolic volume; STEMI: ST-segment-elevation myocardial infarction.

A

Factor	OR	95% CI	*p*-value
**Classical monocytes (0 h)**	**1.001**	**0.998-1.004**	**0.453**
Age	1.032	0.936-1.138	0.522
Sex	0.509	0.025-10.383	0.661
Creatin Kinase	1.000	1.000-1.001	0.311
Troponin-T	1.000	1.000-1.000	0.281

B

Factor	OR	95% CI	*p*-value
**Classical monocytes (6 h)**	**1.003**	**1.000-1.005**	**0.094**
Age	1.055	0.947-1.176	0.330
Sex	0.230	0.011-4.691	0.340
Creatin Kinase	1.000	1.000-1.001	0.155
Troponin-T	1.000	1.000-1.000	0.474

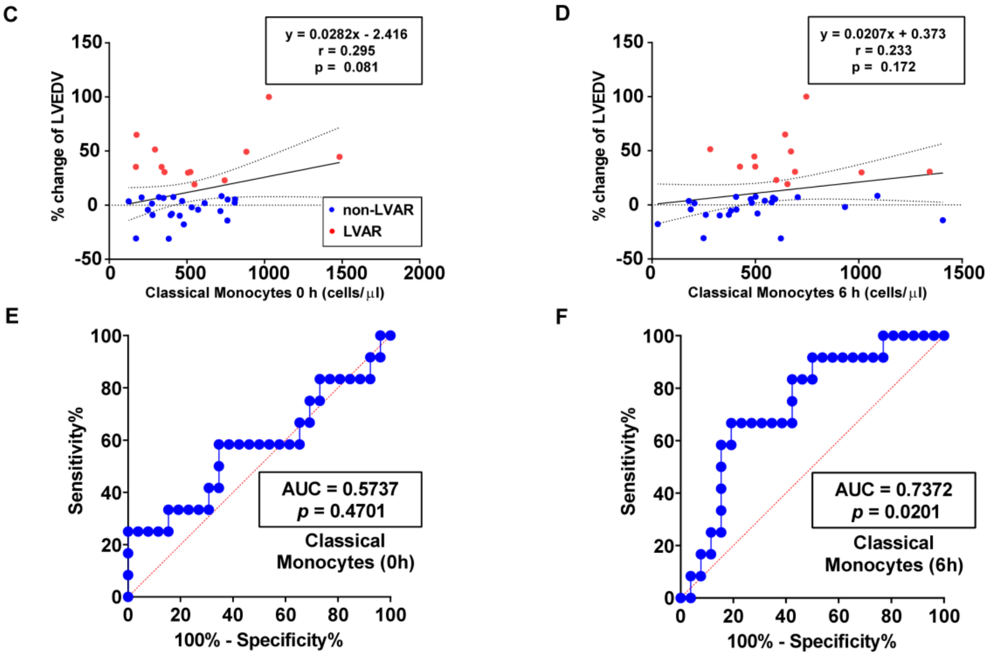

Supplemental Figure 2. Correlation analysis of the level of classical monocytes with LVAR. A, B Multivariate logistic regressions analysis of classical monocytes measured in PB at 0h (**A**) and 6h (**B**), using age, sex, creatine kinase and troponin-T as cofactors. **C, D** Linear regression curves analysis using the percentage of change in LVEDV of non-LVAR (blue dots, n = 26) and LVAR (red dots, n = 12) patients as dependent variable and classical monocytes level at 0h (**C**) or 6h (**D**) as independent factor. **E, F** ROC analysis with the AUC (values given on the graphs) indicating sensitivity and specificity of level of classical monocytes at 0h (**E**) or 6h (**F**) to predict LVAR. Multivariate logistic regression, linear regression and ROC analysis were performed. $p < 0.05$ is considered statistically significant. AUC: area under the curve; CI: confidence interval; LVAR: left ventricle adverse remodelling; LVEDV: left ventricular end-diastolic volume; OR: odds ratio; PB: peripheral blood; ROC: Receiver-operating characteristics.

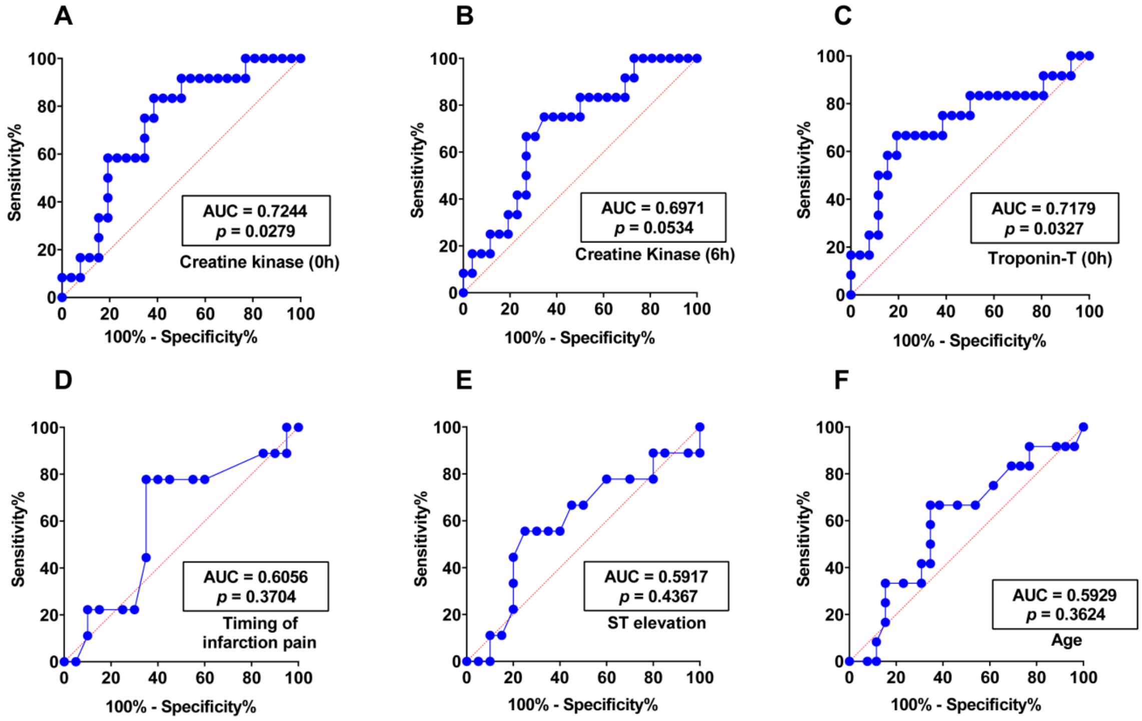

Supplemental Figure 3. ROC curves analysis comparing sensitivity and specificity of different factors as predictors of LVAR. A, F ROC analysis with the AUC (values given on the graphs), indicating sensitivity and specificity of the level of creatine kinase at 0h (**A**) and 6h (**B**), troponin-T (**C**), time of infarction pain (**D**), percentage of ST elevation in the electrocardiogram (**E**) and age (**F**) as predictors of LVAR. ROC analysis was performed. $p < 0.05$ is considered statistically significant. AUC: area under the curve; LVAR: left ventricle adverse remodelling; ROC: Receiver-operating characteristics.

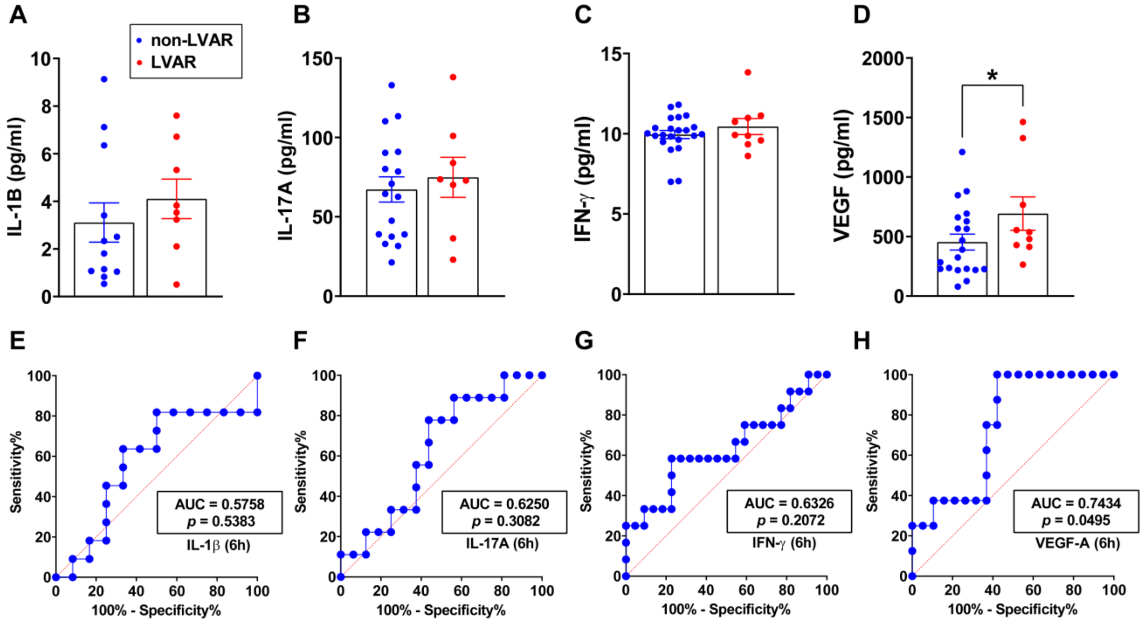

Supplemental Figure 4. Levels of pro-inflammatory cytokines in the serum of STEMI patients measured by ELISA. A, D Bar graphs show level of IL-1β (**A**), IL-17A (**B**), IFN-γ, (**C**) and VEGF (**D**) (pg/ml) in the serum of non-LVAR (n = 19, blue dots) and LVAR (n = 8, red dots) patients, 6h after PPCI. Data are shown as the means ± SEM. **E-H** ROC analysis with the AUC (values given on the graphs), indicating sensitivity and specificity of the level of IL-1β (**E**), IL-17A (**F**), IFN-γ (**G**) and VEGF (**H**) as predictors of LVAR. Mann-Whitney (non-parametric) and T-test (parametric) and ROC analysis were performed. (*) indicates significance at $p<0.05$. AUC: area under the curve; ELISA: enzyme-linked immunosorbent assay; IFN-γ: interferon γ; IL-1β: interleukin 1β; IL-17A: interleukin 17A; LVAR: left ventricular adverse remodelling; PPCI: primary percutaneous coronary intervention; ROC: Receiver-operating characteristics; STEMI: ST-segment-elevation myocardial infarction; VEGF: vascular endothelial growth factor.

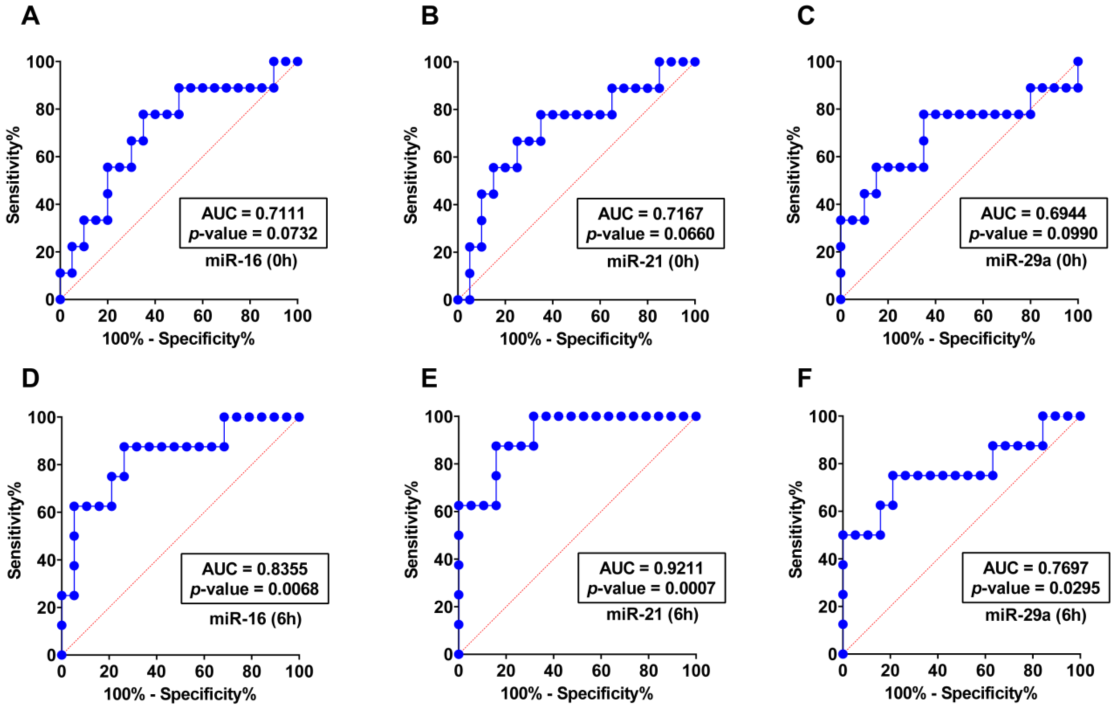

Supplemental Figure 5. ROC analysis of miR-16, miR-21 and miR-29a. A-F ROC analysis with the area under the AUC (values given on the graphs), indicating sensitivity and specificity of the level of expression of miR-16 (**A**)**,** miR-21 (**B**), and miR-29a at 0h (**C**) and at 6h (**D, F**) as predictors of LVAR. ROC analysis was performed. $p < 0.05$ is considered statistically significant. AUC: area under the curve; LVAR: left ventricular adverse remodelling; ROC: Receiver-operating characteristics.

2.2 Supplementary Tables

Cytokine (pg/ml)	0 hour			6 hours			1 month		
	Non-LVAR	LVAR	*p-value*	Non-LVAR	LVAR	*p-value*	Non-LVAR	LVAR	*p-value*
**Eotaxin**	73.13±42.50	91.25±28.70	0.9244	80.10±48.86	88.45±49.58	0.9916	94.18±59.00	85.72±29.48	0.9913
**FGF-basic**	38.73±19.45	54.71±20.12	0.3838	38.95±9.76	47.86±36.60	0.8003	32.95±6.97	25.89±9.84	0.8878
**G-CSF**	52.50±20.07	70.06±36.91	0.5367	60.94±22.57	82.72±35.76	0.3546	64.61±11.45	11.45±2.32	0.8507
**GM-CSF**	23.70±19.52	69.64±23.80	**0.0438***	12.23±26.52	51.45±60.24	0.1686	13.41±24.12	1.49±1.77	0.8773
**IFN-γ**	53.38±20.71	96.46±43.41	0.3037	76.59±38.08	164.65±17.75	**0.0193***	93.72±49.53	52.17±27.26	0.3051
**IL-17**	36.85±17.50	53.63±49.42	0.7356	61.87±29.03	119.50±0.71	**0.0291***	64.35±25.04	44.23±20.28	0.6147
**IL-1β**	1.10±0.55	2.93±2.96	0.147	2.18±0.79	5.74±0.84	**0.005***	2.04±1.52	1.71±0.34	0.9835
**IL-1Ra**	22.26±14.60	75.08±44.15	0.4287	64.34±91.75	68.18±77.28	0.9995	34.14±33.89	32.81±25.17	>0.9999
**IL-4**	6.10±2.00	7.10±1.87	0.9162	9.57±2.62	10.20±4.26	0.9764	9.36±2.75	8.20±1.71	0.8777
**IL-8**	12.52±6.43	12.33±2.08	>0.9999	21.81±15.11	18.33±6.66	0.9204	13.93±3.32	16.67±4.93	0.9602
**IL-9**	62.44±20.93	64.67±44.38	0.9996	118.67±41.06	108.33±62.14	0.9628	112.11±30.69	84.33±30.01	0.5795
**IP-10**	1033±1106	991.33±561.91	>0.9999	1111±1813	1209±1112	0.999	646.44±246.85	1135±289.99	0.8959
**MIP-1α**	3.30±1.03	4.31±2.22	0.563	3.35±0.73	5.04±3.02	0.1569	3.95±0.94	2.66±0.49	0.3638
**MIP-1β**	142.74±85.86	144.82±64.15	>0.9999	224.73±210.45	141.46±75.95	0.6756	126.78±27.22	145.98±51.26	0.9936
**PDGF-BB**	593.07±398.74	1210.82±855.15	0.6756	1364±1055	2267±1825	0.3718	1063±818.90	870.26±619.44	0.9844
**TNF-α**	53.50±9.01	85.62±38.86	0.0503	71.64±10.99	102.85±48.27	0.0593	70.47±11.92	64.33±15.55	0.9506
**VEGF**	6.35±2.83	149.90	-	28.01±16.98	94.34±76.91	**0.0108***	15.09±10.95	52.79±36.07	0.1705

Supplemental Table 1. Levels of pro-inflammatory cytokines in the serum of STEMI patients measured by Bioplex. The table shows level of eotaxin, fibroblast growth factor basic (FGF basic), granulocyte colony stimulating factor (G-CSF), granulocyte-macrophage colony stimulating factor (GM-CSF), interferon γ (IFN-γ), interleukin 17 (IL-17), interleukin 1β (IL-1β), interleukin 1 receptor antagonist (IL-1Ra), interleukin 4 (IL-4), interleukin 8 (IL-8), interleukin 9 (IL-9), inducible protein

10 (IP-10), macrophage inflammatory protein 1α (MIP-1α), macrophage inflammatory protein 1β (MIP-1β), platelet-derived growth factor BB (PDGF-BB), tumor necrosis factor α (TNF-α) and vascular endothelial growth factor (VEGF) (pg/ml) in the serum of patients with LVAR (n=3) or without LVAR (n=10) at 0h, 6h and 1 month after PPCI. Data are shown as the means ± SEM. (*) indicates significance at $p<0.05$. LVAR: left ventricular adverse remodelling; PPCI: primary percutaneous coronary intervention; STEMI: myocardial infarction with ST-segment elevation.

Non-LVAR 6h vs LVAR 6h

	Fold Change	P-value
**IL-1β target of:**		
hsa-miR-21-5p	21,53	0,0277
**IFN-γ target of:**		
hsa-miR-16-5p	5,33	0,0099
hsa-miR-15a-5p	8,02	0,0122
hsa-miR-27b-3p	9,05	0,0143
hsa-miR-27a-3p	6,07	0,0257
hsa-miR-29a-3p	5,36	0,041
**VEGF-A target of:**		
hsa-miR-140-5p	4,05	0,0019
hsa-miR-20a-5p	10,9	0,0075
hsa-miR-93-5p	2,14	0,01
hsa-miR-20b-5p	6,82	0,01
hsa-miR-503-5p	4,1	0,0116
hsa-miR-15a-5p	8,02	0,0122
hsa-miR-126-3p	8,44	0,0141
hsa-miR-195-5p	5,01	0,0186
hsa-miR-106a-5p	6,02	0,0258
hsa-miR-185-5p	7,06	0,0284

hsa-miR-17-5p	4,92	0,0303
hsa-miR-107	4,52	0,0351
hsa-miR-29a-3p	5,36	0,041
hsa-miR-106b-5p	7,81	0,0446

Supplemental table 2. Microarray list of miRNAs targeting IL-1β, IFN-γ and VEGF-A genes. *In silico* analysis of cytokines miRNAs targets analysed in the study by ELISA. miRNAs fold change regulating the expression of the pro-inflammatory cytokines in non-LVAR compared to LVAR patients, 6 hours post-PPCI is shown. ELISA: enzyme-linked immunosorbent assay; IFN-γ: interferon γ; IL-1β: interleukin 1β; LVAR: left ventricular adverse remodelling; miRNAs: microRNAs; PPCI: primary percutaneous coronary intervention; VEGF: vascular endothelial growth factor.

microRNA	Gene ID	Gene symbol	Gene name	Pathway
miR-16-5p	HUMAN|HGNC=393|UniProtKB=Q9Y243	AKT3	RAC-gamma serine/threonine-protein kinase	
	HUMAN|HGNC=9605|UniProtKB=P35354	PTGS2	Prostaglandin G/H synthase 2	
	HUMAN|HGNC=5438|UniProtKB=P01579	IFNG	Interferon gamma	
	HUMAN|HGNC=23366|UniProtKB=Q9BPX5	ARPC5L	Actin-related protein 2/3 complex subunit 5-like protein	
	HUMAN|HGNC=144|UniProtKB=P63261	ACTG1	Actin, cytoplasmic 2	
	HUMAN|HGNC=6180|UniProtKB=Q14643	ITPR1	Inositol 1,4,5-trisphosphate receptor type 1	
	HUMAN|HGNC=9588|UniProtKB=P60484	TEP1	Phosphatidylinositol 3,4,5-trisphosphate 3-phosphatase and dual-specificity protein phosphatase PTEN	
	HUMAN|HGNC=6859|UniProtKB=O43318	MAP3K7	Mitogen-activated protein kinase kinase kinase 7	
	HUMAN|HGNC=1497|UniProtKB=O14936	CASK	Peripheral plasma membrane protein CASK	
	HUMAN|HGNC=6407|UniProtKB=P01116	KRAS	GTPase KRas	
	HUMAN|HGNC=19663|UniProtKB=Q9UBI6	GNG12	Guanine nucleotide-binding protein G(I)/G(S)/G(O) subunit gamma-12	
	HUMAN|HGNC=16852|UniProtKB=O75159	SOCS5	Suppressor of cytokine signaling 5	

	HUMAN|HGNC=7794|UniProtKB=P19838	NFKB1	Nuclear factor NF-kappa-B p105 subunit	
	HUMAN|HGNC=1463|UniProtKB=Q13555	CAMK2G	Calcium/calmodulin-dependent protein kinase type II subunit gamma	
	HUMAN|HGNC=4566|UniProtKB=P62993	GRB2	Growth factor receptor-bound protein 2	
	HUMAN|HGNC=9829|UniProtKB=P04049	RAF1	RAF proto-oncogene serine/threonine-protein kinase	
	HUMAN|HGNC=8591|UniProtKB=Q13177	PAK2	Serine/threonine-protein kinase PAK 2	
	HUMAN|HGNC=6192|UniProtKB=O60674	JAK2	Tyrosine-protein kinase JAK2	
	HUMAN|HGNC=6137|UniProtKB=P17301	ITGA2	Integrin alpha-2	
	HUMAN|HGNC=132|UniProtKB=P60709	ACTB	Actin, cytoplasmic 1	
	HUMAN|HGNC=6204|UniProtKB=P05412	JUN	Transcription factor AP-1	
	HUMAN|HGNC=9954|UniProtKB=Q04864	REL	Proto-oncogene c-Rel	
	HUMAN|HGNC=1974|UniProtKB=O15111	CHUK	Inhibitor of nuclear factor kappa-B kinase subunit alpha	
	HUMAN|HGNC=16833|UniProtKB=O14544	SOCS4	Suppressor of cytokine signaling 6	
	HUMAN|HGNC=10619|UniProtKB=P78556	CCL20	C-C motif chemokine 20	
	HUMAN|HGNC=9401|UniProtKB=Q02156	PRKCE	Protein kinase C epsilon type	

miR-21-5p	HUMAN|HGNC=10637|UniProtKB=P02778	CXCL10	C-X-C motif chemokine 10	INFLAMMATION MEDIATED BY CHEMOKINE AND CYTOKINE SIGNALING PATHWAY
	HUMAN|HGNC=19392|UniProtKB=Q8WXH5	SOCS4	Suppressor of cytokine signaling 4	
	HUMAN|HGNC=4390|UniProtKB=P50148	GNAQ	Guanine nucleotide-binding protein G(q) subunit alpha	
	HUMAN|HGNC=9588|UniProtKB=P60484	PTEN	Phosphatidylinositol 3,4,5-trisphosphate 3-phosphatase and dual-specificity protein phosphatase PTEN	
	HUMAN|HGNC=11364|UniProtKB=P40763	STAT3	Signal transducer and activator of transcription 3	
	HUMAN|HGNC=392|UniProtKB=P31751	AKT2	RAC-beta serine/threonine-protein kinase	
	HUMAN|HGNC=5992|UniProtKB=P01584	IL1B	Interleukin-1 beta	
	HUMAN|HGNC=1608|UniProtKB=P32248	CCR7	C-C chemokine receptor type 7	
	HUMAN|HGNC=16852|UniProtKB=O75159	SOCS5	Suppressor of cytokine signaling 5	
	HUMAN|HGNC=130|UniProtKB=P62736	ACTA2	Actin, aortic smooth muscle	
	HUMAN|HGNC=7794|UniProtKB=P19838	NFKB1	Nuclear factor NF-kappa-B p105 subunit	
	HUMAN|HGNC=7774|UniProtKB=O94916	NFAT5	Nuclear factor of activated T-cells 5	
	HUMAN|HGNC=1602|UniProtKB=P32246	CCR1	C-C chemokine receptor type 1	
	HUMAN|HGNC=1607|UniProtKB=P51684	CCR6	C-C chemokine receptor type 6	

**miR-29a-3p**	HUMAN|HGNC=1461 |UniProtKB=Q13554	CAMK2B	Calcium/calmodulin-dependent protein kinase type II subunit beta	
	HUMAN|HGNC=393| UniProtKB=Q9Y243	AKT3	RAC-gamma serine/threonine-protein kinase	
	HUMAN|HGNC=6155 |UniProtKB=P05107	ITGB2	Integrin beta-2	
	HUMAN|HGNC=1612 |UniProtKB=O00421	CCR6	C-C chemokine receptor-like 2	
	HUMAN|HGNC=5965 |UniProtKB=Q08334	IL10RB	Interleukin-10 receptor subunit beta	
	HUMAN|HGNC=5438 |UniProtKB=P01579	IFNG	Interferon gamma	
	HUMAN|HGNC=9954 |UniProtKB=Q04864	REL	Proto-oncogene c-Rel	
	HUMAN|HGNC=2213 |UniProtKB=P12111	COL6A3	Collagen alpha-3(VI) chain	
	HUMAN|HGNC=9991 |UniProtKB=Q08116	RGS1	Regulator of G-protein signaling 1	
	HUMAN|HGNC=236| UniProtKB=O95622	ADCY5	Adenylate cyclase type 5	
	HUMAN|HGNC=7776 |UniProtKB=Q13469	NFATC2	Nuclear factor of activated T-cells, cytoplasmic 2	
	HUMAN|HGNC=9588 |UniProtKB=P60484	PTEN	Phosphatidylinositol 3,4,5-trisphosphate 3-phosphatase and dual-specificity protein phosphatase PTEN	
	HUMAN|HGNC=392| UniProtKB=P31751	AKT2	RAC-beta serine/threonine-protein kinase	
	HUMAN|HGNC=2212 |UniProtKB=P12110	COL6A2	Collagen alpha-2(VI) chain	

	HUMAN|HGNC=1736|UniProtKB=P60953	CDC42	Cell division control protein 42 homolog	
	HUMAN|HGNC=708|UniProtKB=O15511	ARPC5	Actin-related protein 2/3 complex subunit 5	
	HUMAN|HGNC=436|UniProtKB=P20292	ALOX5AP	Arachidonate 5-lipoxygenase-activating protein	
**miR-16-5p**	HUMAN|HGNC=5438|UniProtKB=P01579	IFNG	Interferon gamma	**INTERFERON-GAMMA SIGNALING PATHWAY**
	HUMAN|HGNC=19382|UniProtKB=O14508	SOCS2	Suppressor of cytokine signaling 2	
	HUMAN|HGNC=16852|UniProtKB=O75159	SOCS5	Suppressor of cytokine signaling 5	
	HUMAN|HGNC=6192|UniProtKB=O60674	JAK2	Tyrosine-protein kinase JAK2	
**miR-21-5p**	HUMAN|HGNC=16833|UniProtKB=O14544	SOCS4	Suppressor of cytokine signaling 6	
	HUMAN|HGNC=19392|UniProtKB=Q8WXH5	SOCS4	Suppressor of cytokine signaling 4	
	HUMAN|HGNC=16852|UniProtKB=O75159	SOCS5	Suppressor of cytokine signaling 5	
	HUMAN|HGNC=16861|UniProtKB=Q9Y6X2	PIAS3	E3 SUMO-protein ligase PIAS3	
**miR-29a-3p**	HUMAN|HGNC=5438|UniProtKB=P01579	IFNG	Interferon gamma	
**miR-16-5p**	HUMAN|HGNC=3326|UniProtKB=P28324	ELK4	ETS domain-containing protein Elk-4	**INTERLEUKIN SIGNALING PATHWAY**
	HUMAN|HGNC=6859|UniProtKB=O43318	MAP3K7	Mitogen-activated protein kinase kinase kinase 7	
	HUMAN|HGNC=6887|UniProtKB=P49137	MAPKAPK2	MAP kinase-activated protein kinase 2	

	HUMAN|HGNC=7553|UniProtKB=P01106	MYC	Myc proto-oncogene protein	
	HUMAN|HGNC=4566|UniProtKB=P62993	GRB2	Growth factor receptor-bound protein 2	
	HUMAN|HGNC=9829|UniProtKB=P04049	RAF1	RAF proto-oncogene serine/threonine-protein kinase	
	HUMAN|HGNC=3942|UniProtKB=P42345	MTOR	Serine/threonine-protein kinase mTOR	
	HUMAN|HGNC=10432|UniProtKB=P51812	RPS6KA3	Ribosomal protein S6 kinase alpha-3	
	HUMAN|HGNC=1784|UniProtKB=P38936	CDKN1A	Cyclin-dependent kinase inhibitor 1	
	HUMAN|HGNC=4617|UniProtKB=P49841	GSK3B	Glycogen synthase kinase-3 beta	
	HUMAN|HGNC=1974|UniProtKB=O15111	CHUK	Inhibitor of nuclear factor kappa-B kinase subunit alpha	
**miR-21-5p**	HUMAN|HGNC=7111|UniProtKB=Q9HBH9	MKNK2	MAP kinase-interacting serine/threonine-protein kinase 2	
	HUMAN|HGNC=10432|UniProtKB=P51812	RPS6KA3	Ribosomal protein S6 kinase alpha-3	
	HUMAN|HGNC=1784|UniProtKB=P38936	CDKN1A	Cyclin-dependent kinase inhibitor 1	
	HUMAN|HGNC=3821|UniProtKB=O43524	FOXO3	Forkhead box protein O3	
	HUMAN|HGNC=11364|UniProtKB=P40763	STAT3	Signal transducer and activator of transcription 3	
	HUMAN|HGNC=392|UniProtKB=P31751	AKT2	RAC-beta serine/threonine-protein kinase	

	HUMAN|HGNC=7553|UniProtKB=P01106	MYC	Myc proto-oncogene protein	
	HUMAN|HGNC=9871|UniProtKB=P20936	RASA1	Ras GTPase-activating protein 1	
	HUMAN|HGNC=6019|UniProtKB=P08887	IL6R	Interleukin-6 receptor subunit alpha	
**miR-29a-3p**	HUMAN|HGNC=393|UniProtKB=Q9Y243	AKT3	RAC-gamma serine/threonine-protein kinase	
	HUMAN|HGNC=5965|UniProtKB=Q08334	IL10RB	Interleukin-10 receptor subunit beta	
	HUMAN|HGNC=10432|UniProtKB=P51812	RPS6KA3	Ribosomal protein S6 kinase alpha-3	
	HUMAN|HGNC=3821|UniProtKB=O43524	FOXO3	Forkhead box protein O3	
	HUMAN|HGNC=392|UniProtKB=P31751	AKT2	RAC-beta serine/threonine-protein kinase	
	HUMAN|HGNC=3796|UniProtKB=P01100	FOS	Proto-oncogene c-Fos	

Supplemental Table 3. *In silico* target genes analysis of miR-16, miR-21 and miR-29a. Genes related to inflammation pathway are shown.

		SCORE RANGE			
**Score**		**1**	**2**	**3**	**4**
**CK**	Creatine kinase 0h (mg/dL)	*from* 271.00 *to* 2071.25	*from* 2071.26 *to* 3871.50	*from* 3871.51 *to* 5671.75	*from* 5671.76 *to* 7472.00
**TnT**	Troponin-T 0h (ng/mL)	*from* 1376.00 *to* 5276.75	*from* 5276.76 *to* 9177.50	*from* 9177.51 *to* 13078.25	*from* 13078.26 *to* 16979.00
**VEGF**	VEGF *(pg/ml)*	*from* 78.75 *to* 337.39	*from* 337.40 *to* 683.25	*from* 683.26 *to* 1029.10	*from* 1029.11 *to* 1374.96
**Int Monocytes**	Intermediate Monocytes (cells/µl)	*from* 24.11 *to* 102.01	*from* 102.02 *to* 179.91	*from* 179.92 *to* 257.81	*from* 257.82 *to* 335.72
**miRNAs**	Hsa-miR-16-5p *(log Fold Change)*	*from* -0.7784 *to* -0.1586	*from* -0.1587 *to* 0.4613	*from* 0.4614 *to* 1.0812	*from* 1.0813 *to* 1.7010
	Hsa-miR-21-5p *(log Fold Change)*	*from* -1.0658 *to* -0.5204	*from* -0.5205 *to* 0.0250	*from* 0.0251 *to* 0.5704	*from* 0.5705 *to* 1.1159
	Hsa-miR-29a-3p *(log Fold Change)*	*from* -0.8199 *to* -0.4301	*from* -0.4302 *to* -0.0402	*from* -0.0403 *to* 0.3496	*from* 0.3497 *to* 0.739487

Supplemental Table 4. Score ranking level for CK, TnT, VEGF, intermediate monocytes and miRNAs. The table shows how scores from 1 to 4 are assigned according to the respective range levels of: CK reported as mg/dL and TnT reported as ng/mL at 0h, and VEGF reported as pg/ml, intermediate monocytes reported as cells/µl, and miRNAs (miR-16, miR-21 and miR-29a expression reported as log fold change) at post-PPCI (6h). CK: creatine kinase; Int Monocytes: intermediate monocytes; miRNAs: microRNAs; PPCI: primary percutaneous coronary intervention; TnT: troponin-T; VEGF: vascular endothelial growth factor.
